# Role of Catestatin in the Cardiovascular System and Metabolic Disorders

**DOI:** 10.3389/fcvm.2022.909480

**Published:** 2022-05-19

**Authors:** Ewa Zalewska, Piotr Kmieć, Krzysztof Sworczak

**Affiliations:** Department of Endocrinology and Internal Medicine, Medical University of Gdansk, Gdansk, Poland

**Keywords:** catestatin, cardiovascular system, hypertension, heart failure, coronary artery disease, immunometabolism, metabolic disorder

## Abstract

Catestatin is a multifunctional peptide that is involved in the regulation of the cardiovascular and immune systems as well as metabolic homeostatis. It mitigates detrimental, excessive activity of the sympathetic nervous system by inhibiting catecholamine secretion. Based on *in vitro* and *in vivo* studies, catestatin was shown to reduce adipose tissue, inhibit inflammatory response, prevent macrophage-driven atherosclerosis, and regulate cytokine production and release. Clinical studies indicate that catestatin may influence the processes leading to hypertension, affect the course of coronary artery diseases and heart failure. This review presents up-to-date research on catestatin with a particular emphasis on cardiovascular diseases based on a literature search.

## Introduction

The sympathetic nervous system is crucial in preserving homeostasis in humans. However, its excessive activity has been recognized to underlie pathologic processes of many cardiovascular diseases (CVDs), which are the leading cause of death globally (1). Among others, upregulated sympathetic nervous system activity has been associated with hypertension (HT), adverse myocardial remodeling, arrhythmias, sudden cardiac death, and overall poor prognosis in patients with heart failure (HF) (2).

Basal and reflex control of the sympathetic activity associated with cardiovascular function occurs in the rostral ventrolateral medulla (3). It sends catecholaminergic projections to the sympathetic preganglionic neurons of the spinal cord. In turn, preganglionic neurons release acetylcholine to activate postganglionic neurons and chromaffin cells of the adrenal medulla, which synthesize and secrete catecholamines (CAs): norepinephrine and epinephrine (stored in sufficient quantities within the cells, which is the reason for a positive chromaffin reaction, and, hence, their name) (3, 4). CAs target α- and β-adrenergic receptors, a family of G protein-coupled receptors. Adrenoceptors are divided into α1, α2, β1, β2, and β3 subtypes. In the smooth muscle cells of blood vessels, there are mainly α1-adrenergic receptors, coupled to stimulatory Gq proteins, which induce constriction by activating phospholipase C. In the heart, the predominant receptor subtype β1 activates the Gs-adenylyl cyclase – adenosine monophosphate – protein kinase A signaling cascade to induce positive inotropic and chronotropic effects (5). α2 receptors are coupled to inhibitory Gi proteins that inactivate adenylyl cyclase and are mainly found in the central nervous system, where their activation lowers arterial blood pressure (BP) (5).

In 1965, Banks and Helle reported that CA secretion from chromaffin granules of the adrenal medulla is associated with the release of soluble proteins (6). In July 1967, Blaschko's group coined the term “chromogranin A” (CgA) for the major component of these proteins (7, 8). In 1988, Simon et al. reported for the first time that proteolytic hydrolysis of CgA, obtained from cultured bovine adrenal medullary chromaffin cells, generated a peptide product that is capable of inhibiting CAs release, a promising and novel mechanism for counteracting the sympathetic outflow (9, 10). However, the exact identity of this functional peptide remained elusive until 1997, when Mahata et al. synthesized 15 peptides spanning ~80% of the entire CgA molecule and demonstrated that only one, bovine CgA_344−364_ [RSMRLSFRARGYGFRGPGLQL], inhibited CA secretion induced by nicotine (11). They named it “catestatin” (Cts) due to its high capacity to suppress the release of CAs (11).

**Figure 1:**
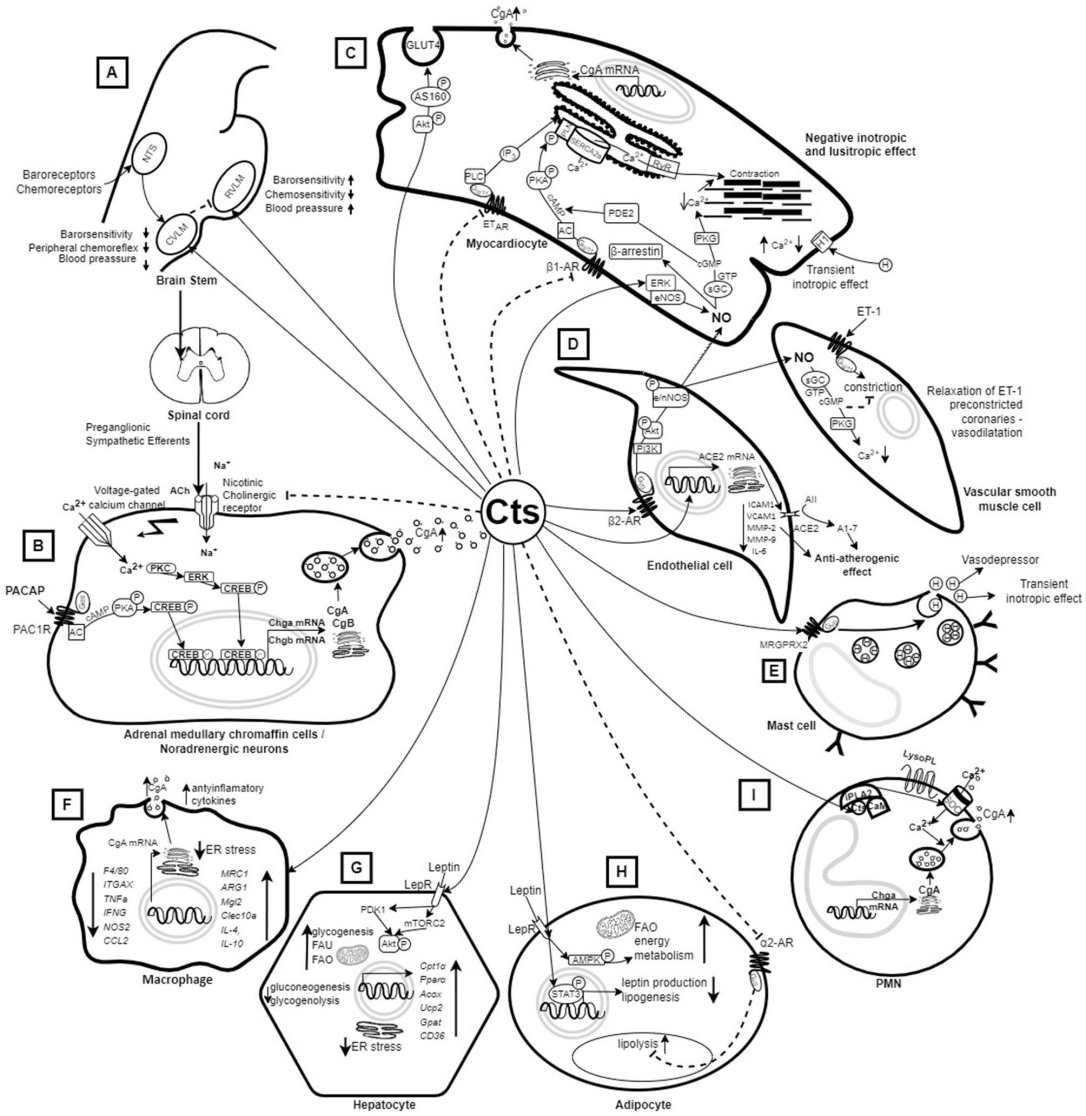
Mechanism of action of catestatin based on *in vitro* and *in vivo* animal studies. **(A)** Injection of Cts into the CVLM or the central amygdala (not shown) of rats decreases sympathetic barosensitivity and attenuates peripheral chemoreflex with consequent hypotension. On the other hand, injection of Cts into the RVLM increases barosensitivity and attenuates chemosensitivity with consequent elevation of blood pressure (14). **(B)** Cts inhibits CA release by binding to nicotinic acetylcholine receptors that block Na^+^ uptake (15) as well as due to PACAP stimulation (13). **(C)** Cts inhibits the PKA/PLN signaling pathway and induces NO synthesis in myocardiocytes, and the released NO reduces cellular Ca^2+^, resulting in decreased cardiac contractility (16) and relaxation of ET-1 preconstricted coronaries (17). Cts also induces glucose uptake and Glut4 translocation (18). **(D)** Cts induces NO synthesis from endothelial cells, and activates ACE2, which has an anti-atherogenic effect (15, 16, 19). **(E)** Cts induces histamine release leading to vasodepression and transient inotropic effect in myocardiocytes (16). **(F)** Treatment with Cts results in polarization of macrophages toward an anti-inflammatory phenotype (20). Macrophages also produce Cts (21). **(G)** Cts up-regulates genes promoting fatty acid oxidation (22) and enhances insulin-induced Akt phosphorylation, which helps in overcoming ER stress and achieving insulin sensitivity (23). **(H)** Cts promotes lipid flux from adipose tissue toward the liver and lowers plasma leptin in Chga-KO mice leading to resensitization of leptin receptors (22). **(I)** PMNs are able to produce and secrete CgA-derived peptides, including Cts, which may penetrate into PMNs and activate the release of innate immune factors (24). A1-7, Angiotensin 1-7; AII, Angiotensin II; AC, Adenylyl cyclase; ACE2, activates angiotensin-converting enzyme-2; Ach, acetylcholine; Acox1, acyl-CoA oxidase 1; Akt, Protein kinase B; AMPK, AMP-activated protein kinase; ARG1, Arginase 1 gene; β1AR, β1 adrenergic receptors; β2AR, β2 adrenergic receptors; Ca^2+^, calcium ions; cAMP, adenosine monophosphate; CAs, catecholamines; CCL2, C-C Motif Chemokine Ligand 2; CD36, cluster of differentiation 36; CgA, Chromogranin A; CgB, Chromogranin B; Chga-KO, Chromogranin knockout; cGMP, cyclic guanosine monophosphate; Cpt1α, Carnitine palmitoyltransferase 1α; CREB, cAMP response element-binding protein; Cts, catestatin; CVLM, caudal ventrolateral medulla; DNL, *de novo* lipogenesis; eNOS, endothelial nitric oxide synthase; ER, endoplasmic reticulum; ERK, extracellular signal-regulated kinas; ET-1, endothelin 1; ETAR, Endothelin receptor type A; ETBR, Endothelin receptor type B; FAO, Fatty acid oxidation; FAU, Fatty acid uptake; Gpat4, lipogenic gene glycerol-3-phosphate acyltransferase; GTP, guanosine-5' triphosphate; H, histamine; ICAM1, Intercellular Adhesion Molecule 1; IFNG, Interferon Gamma gene; IL-4, interleukin 4; IL-6, interleukin 6; IL-10, interleukin 10; iPLA2, calcium-independent phospholipase A2; ITGAX, Integrin Subunit Alpha X; LepR, Leptin receptor; LysoPL, lysophospholipids; MMP-2 Matrix Metallopeptidase 2; MMP-9, Matrix metallopeptidase 9; MRC1, Mannose Receptor C-Type 1 gene; MRGPRX2, Mas-Related G Protein-Coupled Receptor-X2; Na+, sodium; NO, nitric oxide; NOS2, Nitric Oxide Synthase 2; nNOS, neuronal nitric oxide synthase; NTS, Nucleus tractus solitarius; P, phosphor; PACAP, Pituitary adenylate cyclase-activating polypeptide; PAC1R, Pituitary adenylate cyclase-activating polypeptide receptor; PDE2, Phosphodiesterase 2; Pi3K, Phosphoinositide 3-kinase; PKA, Protein kinase A; PKC, protein kinase C; PKG, protein kinase G; PLN, phospholamban; PMNs, Polymorphonuclear neutrophils; Pparα, Peroxisome proliferator-activated receptor-α; RVLM, rostral ventrolateral medulla; RyR, Ryanodine receptor; sGC, soluble guanylyl cyclase; SERCA, Sarcoplasmic reticulum Ca-ATPase; SOC, Store-Operated Calcium Channels; STAT3, Signal Transducer And Activator Of Transcription 3; TAG, Triacylglycerols; TNFa, TNF alpha gene; Ucp2, uncoupling protein 2; VCAM1, vascular cell adhesion molecule 1. Dashed arrow – inhibition; continuous arrow – stimulation.

Cts was initially considered a regulatory peptide that inhibits CA secretion by acting as a noncompetitive mediator of the nicotinic cholinergic stimulation of chromaffin cells and sympathetic neurite outgrowth (11, 12). Further studies showed that Cts inhibits CA release also due to adenylate cyclase-activating polypeptide stimulation and regulates dense core vesicle quanta (13) (Figure 1). Moreover, Cts has emerged as a pleiotropic peptide, which – among others – is involved in the regulation of the cardiovascular and immune system, as well as metabolic homeostasis (14, 17, 25–27).

This review presents up-to-date research on the role of Cts in the cardiovascular system and metabolic disorders contributing to CVDs, and Cts as a putative clinical biomarker. It is based on an electronic literature search of PubMed database performed April 20, 2022, using the key term ‘catestatin’, which yielded 235 results. Papers were included based on screening of titles and abstracts.

## Catestatin Biology

Cts (human CgA_352−372_, bovine CgA_344−364_, and rat CgA_367−387_) is a neuroendocrine peptide that is derived from the proteolytic cleavage of its precursor compound CgA. Human chromogranin A (hCgA) consists of nine dibasic sites and is cleaved by prohormone convertases, namely, cathepsin L, plasmin, and kallikrein, which generates: 1) Cts, which is a 21-amino acid, hydrophobic peptide derived from hCgA's C terminal fragment, 2) a dysglycemic peptide pancreastatin (hCgA250–301), 3) a vasodilating, antiadrenergic, and antiangiogenic peptide vasostatin-1 (hCgA1–76), 4) a peptide that acts as an antigen for diabetogenic CD4+ T-cell clones WE14 (hCgA324–337), and, 5) a proadrenergic peptide serpinin (hCgA411–436) (15).

Cts includes a sequence [SSMKLSFRARAYGFRGPGPQL] that is highly conserved across various species and is flanked by proteolytic cleavage sites (11, 28). Five single-nucleotide polymorphisms have been identified in the hCgA gene *(CHGA)* region expressing Cts: Gly364Ser (rs9658667), Pro370Leu (rs965868), Arg374Gln (rs9658669) (29), Tyr363Tyr (rs9658666), and Gly367Val (rs200576557) (15, 30, 31). Intriguingly, a genome-wide association study identified two loci that affect Cts concentrations in regions that include genes encoding kallikrein and Factor XII; these enzymes participating in a proteolytic cascade (Factor XII activates prokallikrein to kallikrein, which activtes FXII) were shown to generate active compounds from chromogranin A and B cleavage *in vivo* and *in vitro* (32). In particular, kallikrein produced a 12-amino-acid CgA-fragment (CgA361-372) with preserved biological activity of 21-amino-acid Cts (32).

Apart from limiting CA secretion, Cts is a potent inhibitor of the release of other chromaffin cell cotransmitters, including neuropeptide Y, adenosine triphosphate, and CgA; it is widely distributed in secretory granules of the chromaffin cells, diffuse neuroendocrine system, neuronal cells, bone-marrow derived cells, the auditory system, and the heart (20, 24, 33, 34) (Figure 1). In the latter, processing of CgA can be carried out by extracellular proteases on both cardiomyocyte cell membrane and in the extracellular matrix (33, 35). In a rat model, it was shown that the heart generates intracardiac CgA fragments, including locally derived Cts, in response to hemodynamic and excitatory challenges (36). Moreover, recently, a CgA1-373 fragment that encompasses the Cts domain was found to elicit direct cardiac effects both *in vitro* and *ex vivo* (37).

## Catestatin and Regulation of Blood Pressure

### *In vitro* and *in vivo* Animal Studies

*In vitro*, it was shown Cts binds to α, β, δ, and γ subunits of nicotinic acetylcholine receptors (nAChR) with a high affinity binding site on the β subunit near the membrane surface, which blocks sodium ions' uptake, thus inhibiting membrane depolarization, and blocking the influx of calcium ions (Ca^2+^) through voltage-gated calcium channels (38, 39). Inhibition of Ca^2+^ influx suppresses both CA release by exocytosis (all-or-none secretion) and *CHGA* transcription through a pathway involving the activation of protein kinase C and mitogen-activated protein kinase (40) (Figure 1).

Human Cts polymorphic variants were shown to exhibit varying potency of nAChR inhibition *in vitro* using a rat pheochromocytoma cell line and the human receptor (30, 41, 42). One should bear in mind that lower potency of a Cts variant in this respect does not necessarily translate to higher plasma CAs, since in such a case lower desensitization to CA secretion occurs (i.e., an effect due to repeated exposure of the nAChR to Cts). Further, since Cts blood-lowering effect depends partly on the release of nitric oxide (NO), human Cts Gly364Ser variant (see Clinical Studies) was shown to display lower NO-triggering and absent anti-adrenergic activity compared to wild-type peptide (WT-Cts) *in vitro* (31, 43).

This *in vitro* research concerning CA release suppression by Cts is supported by *in vivo* studies with genetically modified rodents. First, knockout of the CgA gene region encoding Cts (Cts-KO) in mice resulted in HT, left ventricular hypertrophy, and elevated CAs, yet, high BP was abated by intraperitoneal injection of exogenous Cts (21). Moreover, this strain exhibited marked cardiac and adrenal macrophage infiltration as well as increased proinflammatory cytokine levels, which may trigger CA release and escalate HT (21). Chlodronate depletion of macrophages, and bone marrow transfer between Cts-KO and WT mice demonstrated that immunosuppression of macrophages by Cts partly underlies its antihypertensive and anti-inflammatory effects (21).

Second, ablation of the CgA gene in another mouse model (Chga-KO) led to – among others – high baseline BP and elevated plasma CAs as well as exaggerated pressor and depressor responses to phenylephrine and sodium nitroprusside (44–46). Exogenous Cts replacement selectively diminished stress-induced increments in BP and HR (45) and restored the sensitivity of high-pressure baroreceptors, i.e., attenuation of both reflex tachycardia (due to sodium nitroprusside-induced hypotension) and reflex bradycardia (following phenylephrine-induced HT) occured (45). This indicates Cts functions efficiently as an antihypertensive peptide even under stressful conditions.

Third, in a genomically humanized mice strain expressing insufficient hCgA amounts, increased plasma CA levels as well as elevated systolic and diastolic BP were recorded in comparison to WT controls and a strain with sufficient hCgA expression (47).

However, Kennedy et al. showed Cts did not affect plasma norepinephrine levels and actually significantly (11-fold) increased those of epinephrine following electrical stimulation of male Sprague-Dawley rats, although it did reduce BP increases (even with anti-adrenergic pretreatment) (48). The possible explanation of these results, which contrast with findings from *in vitro* and Cst- and CgA-knockout studies, is that epinephrine release was compensatory to effects triggered by Cts in acute injury by electric stimulation, which could not have been observed *in vitro* (due to study limitations) and in KO mice owing to significant genetic alterations. Mitigation of pressor responses by Cts in the study by Kennedy et al. was attributed to histamine release: it increased 21-fold within 2 min of Cts injection, and the BP lowering effect was abolished by hydroxizine pretreatment, which was also the case for epinephrine elevation (48). Krüger et al. confirmed this hypothesis *in vitro*: Cts lead to histamine release from rat mast cells *via* a peptidergic pathway – by activating the G protein, because this effect was suppressed by the pertussis toxin, a Gi/Go inactivator (49) (Figure 1).

Furthermore, although plasma Cts concentrations gradually increased with the progression of HT in spontaneously hypertensive rats, exogenous Cts reduced HR (indicating anti-sympathetic activity) (50), as well as ameliorated vascular, renal and cardiac proliferation (51). *In vitro*, Cts was also reported to act as a potent inhibitor of isoproterenol- and endothelin-1-mediated activities in the frog (52) and rat heart (53). Angelone et al. showed that WT-Cts increased heart rate and decreased left ventricular pressure, rate-pressure product, and, both positive and negative left ventricular contractility. The authors suggested that these negative inotropic and lusitropic effects of WT-Cts may contribute to its hypotensive action (53) (Figure 1).

Concerning the central nervous system, Cts plays an important role in cardiorespiratory control (14). Gaede et al. showed that Cts antagonizes both nAChR and β-adrenergic receptors involved in cardiovascular regulation using urethane-anesthetized, vagotomized rats. Cts mitigated the hypertensive effect of nicotine and prevented increased splanchnic sympathetic activity caused by isoproterenol, a non-selective β adrenergic receptor agonist (54). The results of the study indicate Cts may affect BP levels in HT (54). Further, depending on the region of the medulla, Cts exerted sympathoexcitatory or cholinergic effects (3, 55, 56). Injection of Cts into the rostral ventrolateral medulla (a key site for BP control in the brain stem) resulted in increased barosensitivity and attenuation of chemosensitivity with consequent BP elevation (3). On the other hand, injection of Cts into the caudal ventrolateral medulla (55) and the central amygdala (56) (both contain inhibitory neurons of the rostral ventrolateral medulla) of rats resulted in decreased sympathetic barosensitivity and attenuation of the peripheral chemoreflex with consequent hypotension (Figure 1).

Effects of Cts based on *in vitro* and *in vivo* experimental studies are presented in Figure 1. In summary, Cts may inhibit CA release from chromaffin cells and noradrenergic neurons (11) and induces desensitization of CAs (57); it also exhibits a potent vasodilatory effect mediated – at least in part – by histamine release (48, 49). Moreover, negative inotropic and lusitropic effects of Cts may lower BP (53). Finally, Cts plays a role in central cardiorespiratory control (3, 56). Taken together, experimental studies point to Cts as a novel regulator of BP.

### Clinical Studies

On the one hand, similarly to *in vivo* studies on rodents, it was shown that infusion of Cts into the dorsal hand veins of normotensive individuals, after pharmacologic venoconstriction with phenylephrine, resulted in a dose-dependent vasodilation, which was predominantly observed in female subjects (58). On the other, clinical studies generated controversies regarding the association between Cts levels and primary HT.

O'Connor et al. showed that normotensive offspring of patients with HT had significantly lower Cts concentrations compared to normotensive participants with a negative family history of HT (1.32 ± 0.038 vs. 1.5 ± 0.076 ng/mL, *p* = 0.024). Low plasma Cts levels predicted enhanced pressor response to a sympathoadrenal stressor (59). Therefore, reduced Cts levels were postulated to predispose to the development of HT, although categorization by BP status (normotensive vs. hypertensive) did not reveal differences in plasma Cts (1.36 ± 0.03 vs. 1.26 ± 0.06 ng/mL, *p* = 0.27) (59). In line with the result, Durakoglugil et al. showed that the difference in Cts concentrations between previously untreated hypertensive patients and healthy controls was insignificant after adjusting for age, gender, height, and weight (60). However, in another study by the O'Connor group, in a larger cohort (452 normotensives, 215 primary hypertensives), Cts was significantly reduced in HT patients (1.47 ± 0.06 1.26 vs. 1.26 ± 0.08; *p* = 0.036) (61). In contrast, Meng et al. showed that Cts was significantly higher in patients with essential HT than normal controls (1.19 ± 0.74 vs. 1.53 ± 0.72 ng/mL, *p* < 0.01) but found no correlation between Cts and the degree of HT (1.56 ± 0.59 vs. 1.42 ± 0.59 vs. 1.57 ± 0.76 ng/mL, *p* > 0.05) (62). They also suggested Cts level may serve as a prognostic factor for complications of HT, as patients with left ventricular hypertrophy had lower Cts-to-norepinephrine ratios than those without (3.63 ± 1.62 vs. 2.76 ± 0.86, *p* < 0.01) (62). In order to explain the contradictory results concerning Cts levels in normo- and HT, it was hypothesized that Cts decreases in prehypertension, however, sympathetic nervous system activity increases as HT progresses, which contributes to compensatory Cts elevation. Consequently, HT develops when elevated Cts no longer inhibits CA oversecretion (62).

Cts genetic variants exert varying effects on BP (16). The Gly364Ser variant was associated with lower diastolic BP than the Gly364Gly variant (wild type) in two Caucasian hypertensive groups of European ancestry and ca. 1.8% variance of population diastolic BP was attributable to Cts single nucleotide polymorphism (63). Conversely, in Asian populations, Ser-364 allele carriers displayed elevated systolic (up to ≈8 mm Hg; *p* = 0.004) and diastolic (up to ≈6 mm Hg; *p* = 0.001) BP (Indian) (31) as well as systolic BP, pulse pressure and arterial stiffness (Japanese) (64). In line with these, in a normotensive Indian population, Ser-364 allele carriers exhibited much lower plasma CA - by about 40% (consistent with its diminished nAChR desensitization-blocking effect *in vitro*) - than Gly364Gly carriers (30), yet, BP was similar in subjects with different alleles. These contradictory results underscore the necessity of genetic association studies for ethnically different populations.

Processing of CgA to Cts by endoproteolytic enzymes may be involved in the pathogenesis of HT. O'Connor et al. showed that CgA was increased by 117% (*p* < 0.001) in HT patients, whereas Cts reduced by 15% (*p* = 0.036), which suggests diminished conversion of CgA to Cts in HT (normotensives' CgA/Cts ratio of 4.5 ± 0.2 vs. 5.9 ± 0.4 in HT patients, *p* = 0.005) (61). Fung et al. demonstrated that in women compared to men higher plasma Cts (1.30 ± 0.033 vs. 1.14 ± 0.27 nM, *p* < 0.001) combined with lower CgA precursor concentrations (3.89 ± 0.15 vs. 4.65 ± 0.33 nM, *p* = 0.006) may be associated with decreased processing of CgA to Cts in the latter (female vs. male Cts/CgA ratio: 26.3 ± 0.006 vs. 23.1 ± 0.006), which predisposes to HT (58).

Moreover, Biswas et al. showed that *CHGA* variants undergo differential processing to produce Cts in the presence of the endoproteolytic enzyme plasmin (65). Their study indicates that less efficient processing of the Pro370Leu protein (product of one of *CHGA* variants) can contribute to the prevalence of CVDs (65).

CgA variants may also impact target organ damage in HT: black people suffering from end stage renal disease exhibit a common *CHGA* variant (3′-UTR C+87T), which decreased reporter gene expression and subsequently lead to lower Cts levels in this population (2.10 ± 0.88 vs. 3.23 ± 0.29 ng/mL, *p* = 0.01) (66).

Clinical studies concerning Cts in HT are presented in Table 1. To summarize, Cts was shown to decrease during prehypertension (59), however, the progression of elevated BP was associated with a compensatory increase in its concentration (62). Moreover, the development of HT may well be connected with diminished conversion of CgA to Cts (58, 61, 65) and depends on different variants of Cts exerting varying effects on BP (16, 63).

**Table 1:** Clinical studies concerning catestatin.

**References**	**Study participants**	**Main results**
**Catestatin and hypertension**		
O'Connor et al. (59)	40 normotensives with positive HT family history, 176 normotensives without HT family history, 61 patients with HT	Offspring of HT patients had lower Cts than normotensives without HT family history: 1.32 ± 0.038 vs. 1.5 ± 0.076 (*p* = 0.024) Plasma Cts was not different in normotensives vs. hypertensives (1.36 ± 0.03 vs. 1.26 ± 0.06 (*p* = 0.27)
O'Connor et al. (61)	452 normotensives, 215 patients with HT	Cts was reduced by 15% in hypertensives patients (*p* = 0.036), whereas CgA was increased by 117% (*p* < 0.001); the ratio of CgA/Cts was thus increased by 31% (*p* = 0.005), implying decreased conversion of CgA to Cts in PH.
Salem et al. (66)	Black patients with HT and ESRD (*n* = 150) and black controls (*n* = 58)	ESRD patients had lower Cts: 2.10 ± 0.88 vs. 3.23 ± 0.29 (*p* = 0.01)
Meng et al. (62)	136 HT patients (109 with and 27 without LVH 27) and 61 healthy controls	Cts was higher in hypertensives vs. controls: 1.19 ± 0.74 vs. 1.53 ± 0.72 (*p* < 0.01) There was a non-significant trend toward lower Cts in HT patients with LVH than those without: 1.55 ± 0.7 vs. 1.4 ± 0.53 (*p* > 0.05).
**Catestatin and coronary artery disease**		
Wang et al. (67)	50 STEMI patients and 25 non-CAD control patients	STEMI patients had lower plasma Cts on admission than controls: 16.5 ± 5.4 vs. 21.4 ± 6.4 (*p* < 0.01), increased on day 3 to 30.7 ± 12.2 (*p* < 0.01), and on day 7 decreased to levels below than admission (13.8 ± 5.3, *p* < 0.01) in MI
Meng et al. (68)	52 healthy controls 58 STEMI patients, 31 of whom were assessed by echocardiography 3 months later to reveal 7 cases with LVR and 24 without LVR	Plasma Cts on admission higher in patients with AMI than in controls: 1.00 (0.66–1.50) vs. 0.84 (0.56–1.17) (*p* < 0.05) Mean Cts higher on day 3: 1.12 (0.76–1.70) vs. 1.00 (0.66–1.50) (*p* < 0.01) and on day 7.: 1.32 (0.81–1.73) ng/ml vs. 1.00 (0.66–1.50) (*p* < 0.01) Patients with LVR 3 months after AMI vs. those without had higher Cts: -on admission: 2.02 (1.14–5.87) vs. 0.94 (0.39–1.66) (*p* = 0.001), -on day 3: 2.47 (1.10–5.95) vs. 1.16 (0.31–1.95) (*p* = 0.006), and, -on day 7 after STEMI: 3.08 (0.41–6.77) vs. 1.20 (0.30–3.73) (*p* = 0.021)
Liu et al. (69)	30 healthy controls; 15 SAP, 47 UAP, 22 NSTEMI, and 36 STEMI patients	Plasma Cts was higher in CAD patients than controls: 0.41 ± 0.14 vs. SAP patients: 0.72 ± 0.50 (*p* < 0.05) UAP patients 0.88 ± 0.58 (*p* < 0.05) NSTEMI patients 1.05 ± 0.48 (*p* < 0.05) STEMI patients 1.31 ± 0.91 (p < 0.05)
Pei et al. (70)	STEMI patients with MA (*n* = 61) STEMI patients without MA (*n* = 64)	Plasma Cts was higher in patients with STEMI complicated by MA compared with those without MA: 0.083 ± 0.011 vs. 0.076 ± 0.007 (*p* < 0.001)
Zhu et al. (71)	30 non-CAD controls (n = 30) 100 AMI patients including 74 with adverse events on follow-up and 26 without	Cts lower in MI patients on admission vs. controls: 16.7 ± 5.4 vs. 21.8 ± 6.3 (*p* < 0.0001), higher on day 3 at 30.9 ± 12.1 (*p* < 0.0001 vs. admission), and again lower on day 7: 13.9 ± 5.2 (*p* = 0.0003 vs. admission) Cts on admission and day 3 in the adverse events group (19.4 ± 6.7 ng/ml, 44.6 ± 13.0, respectively) higher than in the non-adverse events group (15.8 ± 4.5 (*p* = 0.003), 26.1 ± 7.5 (*p* < 0.0001), respectively).
Xu et al. (72)	38 patients with CTO and 38 controls	Cts higher in CTO patients than in controls 1.97 ± 1.01 vs. 1.36 ± 0.97 (*p* = 0.009)
Zhu et al. (73)	72 STEMI patients and 30 control patients without CAD on imaging	Patients with Cts level above median at day 3 (28.71 ng/ml) developed worse ventricular function during the 65 months follow-up (*p* < 0.0001).
Xu et al. (74)	46 STEMI patients, 89, 35 control patients without CAD on imaging	Cts in patients with STEMI (0.80 ± 0.62) and UAP (0.99 ± 0.63) lower than in controls (1.38 ± 0.98; *p* = 0.001).
Kojima et al. (75)	25 CAD patients: 20 with AMI and 5 with UAP; controls: 20 non-CAD patients with mild hypertension and 13 healthy volunteers	Plasma Cts levels were lower in CAD patients (2*) than in non-CAD patients (4*; *p* ≤ 0.05).
Chen et al. (19)	204 healthy volunteers 224 CAD patients	CAD patients had lower serum Cts than controls: 1.14 (1.05–1.24) vs. 2.15 (1.92–2.39); *p* < 0.001, and the levels decreased in a stepwise manner with increasing number of diseased vessels: 1.95 (1.83–2.07) vs. 1.57 (1.42–1.73) vs. 1.13 (1.00–1.27), *p* < 0.001 (for 1, 2, and 3 vessels, respectively)
**Catestatin and heart failure**		
Zhu et al. (76)	300 moderate to severe HF patients:−108 in stage A; 76 in Stage, 116 - Stage C	Cts decreased with higher HF stages and there was a significant difference between stage A and B: 21.29 ± 7.10 vs. 14.61 ± 4.69 (*p* < 0.05).
Liu et al. (77)	172 controls 228 HF patients in NYHA class I – IV	Plasma Cts increased with higher classes, NYHA class III and class IV patients had higher Cts levels than controls: 0.848 (0.664–1.260); 1.54 (0.856–2.432), respectively vs. 0.696 (0.504–0.883) (*p* < 0.05). NYHA class I and class II patients had similar Cts to controls: 0.612 (0.52 −0.844); 0.722 (0.532–1.112), respectively vs. 0.696 (0.504–0.883) (*p* > 0.05)
Peng et al. (78)	Cohort of 202 HF patients followed-up for a median of 52.5 months: 143 survived, 59 died – 49 for cardiac causes	Plasma Cts was higher in non-survivors both for all and cardiac causes 1.06 (0.66–1.82) and 1.18 (0.69–1.83), respectively vs. 0.75 (0.58–1.12) in survivors (*p* ≤ 0.005)
Wołowiec et al. (79)	Upon a follow-up of 24 months out of 52 HFrEF patients 11 reached the composite endpoint (CE) of unplanned hospitalization and all-cause death 24 healthy volunteers served as controls	Cts lower in HFrEF patients who reached a CE than in those who did not – both before and after exertion: 14.23 (11.05–15.82) vs. 16.86 (14.25–19.46) (*p* = 0.03) and 4.81 (2.20–6.25) vs. 7.82 (5.81–63.48) (*p* = 0.002), respectively; Cts in patients similar to controls: before exertion 15.95 (13.89–18.81) vs. 16.6 (14.75–22.20) (*p* = 0.12), after: 7.04 (4.97–11.08) vs. 9.26 (6.11–140.23) (*p* = 0.13)
Borovac et al. (80)	96 HF patients hospitalized due to an acute worsening of HF, 6 did not survive	Cts was significantly higher among non-survivors than survivors: 19.8 (9.9–28) vs. 5.6 (3.4–9.8) (*p* < 0.001)
**Catestatin and other diseases affecting the cardiovascular system**		
Sun et al. (81)	330 controls; 329 hemodialysis patients followed-up for 36 months – 29 died for cardiac and 28 for non-cardiac causes	Cts higher in hemodialysis patients (1.9 ± 0.3) vs. controls (1.2 ± 0.2), *p* < 0.001 Cts higher (2.2 ± 0.1) in patients who died for cardiac causes than in survivors (1.8 ± 0.2) and non-cardiac death non-survivors 1.8 ± 0.3), *p* < 0.001
Izci et al. (82)	97 controls 160 APE patients: 72 with sPESI ≥ 1 and 88 <1	Plasma Cts higher in APE patients than controls: 27.3 ± 5.7 vs. 17.5 ± 6.1 (*p* < 0.001); Cts higher in patients with sPESI ≥ 1 than with sPESI <1: 37.3 ± 6.1 vs. 24.2 ± 5.3 (*p* < 0.001)
Tüten et al. (83)	100 women with preeclampsia 100 women with uncomplicated pregnancy as controls.	Plasma Cts was significantly increased in the preeclampsia patients compared to the controls: 0.29 ± 0.096 vs. 0.183 ± 0.072 (*p* < 0.001)
Liu et al. (84)	260 healthy workers	Plasma CgA-to-catestatin ratio correlated with effort, reward (negatively), overcommittment, and efford-reward imbalance: r = 0.218, −0.249, 0.275, and 0.279, respectively, *p* < 0.001 for all
**Catestatin and metabolic syndrome**		
Simunovic et al. (85)	92 obese subjects (BMI Z score >2), age 10-18; 39 controls	Lower plasma Cts concentrations in obese subjects compared to controls: 10.03 ± 5.05 vs. 13.13 ± 6.25 (*p* = 0.004); lower Cts in the subgroup of obese patients with MS: 9.02 ± 4.3 vs. 10.54 ± 5.36 vs. 13.13 ± 6.25 (*p* = 0.008). Cts negatively correlated with DBP (r = −0.253, *p* = 0.014), HOMA-IR (r = −0.215, *p* = 0.037) and hsCRP (r = −0.208, *p* = 0.044).
Kim et al. (86)	85 subjects with mild OSA, 26 with moderate-to-severe OSA, 102 were controls, mean age 7.7 ± 1.4 years	Children with OSA have reduced plasma Cts levels (Log Cts in moderate-to-severe OSA: 0.12 ± 0.22 vs. mild OSA: 0.23 ± 0.20 vs. controls: 0.28 ± 0.19; differences among three groups: *p* < 0.01). Cts levels were inversely correlated with AHI (r = −0.226; *p* < 0.01) and with mean arterial BP level (r = −0.184; *p* < 0.05).
Borovac et al. (87)	78 OSA patients; 51 controls	Plasma Cts higher in OSA patients compared to controls: 2.9 ± 1.2 vs. 1.5 ± 1.1 (*p* < 0.001). In OSA patients Cts correlated with neck circumference (r = 0.318, *p* < 0.001; β = 0.384, *p* < 0.001) and HDL cholesterol (r = −0.320, *p* < 0.001; β = −0.344, *p* < 0.001).

Catestatin concentrations are given in ng/mL and the results are shown as median (interquartile range) or mean ± standard deviation; AHA, American Heart Association; AMI, acute myocardial infarction; APE, Acute pulmonary embolism; AWHF, acute worsening of heart failure; BP, blood preassure; CAD, coronary artery disease; CD, Crohn's disease; CE, composite endpoint including unplanned hospitalization and death for all causes; CPET, Cardiopulmonary Exercise Testing (6-minut walk test); Cts, catestatin; CTO, chronic total occlusions; DBP, diastolic blood pressure; ERI, effort, reward imbalance; ESRD, end stage renal disease; HDL, high-density lipoprotein; HF, heart failure; HFrEF, Heart Failure with Reduced Ejection Fraction; HOMA-IR, homeostatic model assessment of insulin resistance; hsCRP, high sensitivity C-reactive protein; HT, hypertension; IBD, inflammatory bowel diseases; LVH, left ventricular hypertrophy; LVR, left ventricular remodeling; MA, malignant arrhythmia; MS, metabolic syndrome; NSTEMI, non-ST segment elevation myocardial infarction; NYHA, New York Heart Association; OSA, obstructive sleep apnea; SAP, stable angina pectoris; STEMI, acute ST- segment elevation myocardial infarction; sPESI, simplified PESI; UAP, unstable angina pectoris; UC, ulcerative colitis. *Mean value (standard deviation was not provided).

## Cardiac Function of Catestatin

### *In vitro* and *in vivo* Animal Studies

Cts induces NO synthesis from endothelial cells and cardiomyocytes (43, 53). In endothelial cells, NO is acquired from both: (1) the endothelin receptor B – endothelial nitric oxide synthase (eNOS) – NO, and (2) the protein kinase B (Akt) – eNOS/neuronal nitric oxide synthase (nNOS) – NO pathways. In cardiomyocytes, NO release results from the extracellular signal-regulated kinase – eNOS – NO pathway (53) (Figure 1). NO reduces inotropism and lusitropism of cardiomyocytes through various pathways (52, 53, 88, 89) (Figure 1). It was demonstrated *in vitro* using frog (52), eel (89), and rat (90) heart preparations that higher NO generation due to Cts ameliorates the Frank-Starling response, which is a compensatory mechanism to maintain adequate heart function in response to changes in venous return (90). Moreover, Cts exerts counterregulatory action against β-adrenergic and endothelin-1 stimulation pointing to Cts as a novel, beneficial cardiac modulator (53) (Figure 1).

In line with this research, *in vitro*, Cts attenuated norepinephrine-mediated hypertrophic responses in H9c2 cardiac myoblasts and at 10-25 nM signaling was moderated primarily by β1/2-adrenoceptors (91). Interestingly, Bassino et al. observed that a low (5 nM) WT-Cts concentration reduced β-adrenergic stimulation in bovine aortic endothelial cells, although it exhibited no significant effect on myocardial contractility. Higher concentrations (10–50 nM) of Cts induced a transient positive inotropic effect followed by a negative antiadrenergic effect (43). The transient positive effect was probably related to histamine release from mast cells induced by Cts. Histamine exerts a positive inotropic effect on rat ventricular myocardium and H1 histamine receptor antagonist mepyramine blocked the positive effect induced by higher doses of WT-Cts (10 nM). In turn, WT-Cts at a minimal concentration (5 nM) reduced isoproterenol-induced enhancement of papillary muscle contractility via H1 receptor activation without altering basal contractile ability (43).

Several studies point at a cardioprotective role of Cts in ischemia/reperfusion (I/R) injury of the heart in rodent models. It was apparent for Cts pretreatment (25 nM, 50 nM, and 100 nM infused for 15 min before ischemia) (92) and chronic administration of Cts after MI (0.25 mg/kg/12 h injection intraperitoneally 24 h after AMI for 28 days) (67). Further, infusion of a Cts dose of 75 nM for 20 min at the beginning of reperfusion significantly reduced infarct size, limited post-ischemic contracture, and improved recovery of developed left ventricular pressure (93–96). Protection due to Cts in I/R-injured myocardium was related to salvage of oxidative-stress-induced apoptosis by activation of the β2-adrenergic receptor, and PKB/Akt pathway (96). It must be highlighted that infusion of a higher dose WT-Cts (100 nM) for 120 min during reperfusion caused deleterious effects and did not activate Akt (97). Interestingly, the Gly364Ser variant of Cts at a higher dose (100 nM for 120 min) lowered infarct size, which is probably associated with lower inhibitory activity on the nicotinic cholinergic receptor of the Cts-Gly364Ser variant (97). It is worth underlining that cardioprotection achieved by ischemic preconditioning in wild-type mice was absent in Cts-KOs (21).

Modulation of cardiac glucose metabolism is another cardioprotective mechanism of Cts, in particular, improvement of glucose uptake early during post-ischemic reperfusion (18). In physiological conditions, cardiomyocyte metabolism depends mainly on fatty acid oxidation, although the heart shifts toward glucose metabolism in response to ischemia to ameliorate myocardial contractile efficiency. In isolated rat cardiomyocytes, Cts induced Akt and AS160 phosphorylation and significantly enhanced glucose uptake (18), which may be crucial for recovery of contractile function during post-ischemic reperfusion (98).

A recent study involving an *in vivo* and *in vitro* approach investigated Cts in acute pulmonary embolism (99). Its administration in mice with this pathology increased survival as well as augmented thrombus resolution by attenuating endothelial inflammation (99).

In summary, based on *in vitro* and *in vivo* animal models, it was demonstrated that Cts exhibits a potential cardioprotective effect by acting directly as a cardiodepressing peptide through multiple signaling pathways (52, 53, 88–90), it may also reduce apoptosis of cardiomyocytes induced by oxidative stress (67, 92–96), and is beneficial in acute pulmonary embolism (99). However, more studies are needed in these areas, in particular, regarding Cts in I/R injury of the heart (67, 92–97).

### Clinical Studies

#### Coronary Artery Disease

Wang et al. (100) were the first to investigate Cts in patients with coronary artery disease (CAD) by measuring its plasma concentration in 58 acute myocardial infarction (AMI) patients on admission, day 3 and day 7, and in 25 control subjects who were admitted to the same hospital for atypical chest pain but with normal coronary arteries confirmed by coronary angiography. Compared with controls (21.4 ± 6.4 ng/ml), plasma Cts concentrations were significantly lower on admission due to AMI (16.5 ± 5.4 ng/ml, *p* < 0.01), higher on the third day (30.7 ± 12.2 ng/ml, *p* < 0.01) and again lower on day 7 (13.8 ± 5.3 ng/ml, *p* < 0.01).

Zhu et al. (71) and Xu et al. (74) obtained results consistent with the above: compared to controls, Cts was lower on admission due to AMI, it increased significantly on the third day of hospitalization, but decreased 1 week following AMI. In line with these studies, Kojima et al. showed in small samples of patients that Cts was significantly lower in patients with AMI or unstable angina pectoris than in non-CAD patients (75).

However, Meng et al. (68) and Liu et al. (69) showed that plasma Cts levels at the time of admission were significantly higher in patients with AMI compared to healthy controls. The discrepancy between studies may result from different control groups, which included patients with chest pain without CAD on imaging in studies by Wang et al. (100), Zhu et al. (71) and Xu et al. (74) vs. healthy volunteers in studies by Meng et al. (68) and Liu et al. (69).

In addition, Chen et al. demonstrated serum Cts levels were lower in stable angina pectoris (SAP) patients compared to healthy controls (median (inter-quartile range) 1.14 (1.05–1.24) ng/mL vs. 2.15 (1.92–2.39) ng/mL, *p* < 0.001). Moreover, a stepwise decrease in serum Cts was found when classifying CAD patients according to the number of diseased vessels (19). However, Liu et al. showed that SAP patients (*n* = 15) had significantly higher Cts levels compared to controls (0.41 ± 0.14 ng/mL vs. 0.72 ± 0.50 ng/mL, *p* < 0.05) (69). Divergent findings are difficult to account for, perhaps these depend on disease symptoms in patients of the latter study (Cts increases as CAs are released when pain occurs); small SAP patient sample size in the study by Liu et al. could have biased the results (69).

Further, Xu et al. demonstrated that mean plasma Cts in coronary artery chronic total occlusion patients, who underwent coronary angiography or percutaneous coronary intervention for the first time, were significantly higher than in patients with chest pain but with normal coronary arteries (1.97 ± 1.01 vs. 1.36 ± 0.97 ng/ml, *p* = 0.009) (72).

Of the 58 AMI patients mentioned above, Meng et al. studied 31 by echocardiography 3 months after AMI onset, and diagnosed 7 with left ventricular remodeling (LVR). Interestingly, these patients had significantly higher plasma Cts levels on admission, day 3, and day 7 than those without LVR (68). In line with Meng et al., Dan Zhu et al. found that AMI patients, whose Cts level exceeded the median at day 3 of hospitalization (28.71 ng/ml), exhibited worse left ventricular function in echocardiography 65 months after AMI (73).

In another study performed by Dan Zhu et al., adverse events occurred more frequently in the 65-month follow-up in patients whose Cts level on day 3 exceeded the median as well as in those whose ratios of Cts on day 7 to day 3 were below the median (71). Moreover, plasma Cts level turned out to be an independent prognostic factor for malignant arrhythmia (MA) after AMI, and was significantly higher in patients with AMI complicated by MA (70). However, Xu et al. showed no significant differences in major adverse cardiovascular events, including death from cardiovascular causes, recurrent AMI, or hospital admission for HF, or revascularization between patients with high and low Cts concentrations (72).

#### Heart Failure

Concerning HF, Dan Zhu et al. showed that the higher the severity stage of HF (according to the American Heart Association, AHA), the lower the Cts level, and that Cts might be a better predictive factor for stage B HF than brain natriuretic peptide, a marker commonly used in clinical practice for HF diagnosis and severity assessment (76). However, according to a study by Liu et al., plasma Cts levels were increasingly higher in patients with growing severity of the New York Heart Association (NYHA) HF classes I to IV, and NYHA class III and IV patients exhibited significantly higher plasma Cts levels than controls, NYHA class I, and class II subjects (77). Seemingly contradictory results may be a consequence of patient enrollment criteria: Zhu et al. recruited asymptomatic chronic patients with stage C HF (76), while NYHA IV patients had resting dyspnea (77). As mentioned before, symptoms that trigger CA release can cause a compensatory increase in Cts levels.

Peng et al. demonstrated that Cts was an independent risk factor for all-cause death (hazard ratio 1.84 (95% CI: 1.02–3.32, *p* = 0.042)) and cardiac death [hazard ratio 2.41 (95% CI: 1.26–4.62, *p* = 0.008)] during a median 52.5-month follow-up (78). In line with their results, in the CATSTAT-HF Study, Cts was found to be an independent and significant predictor of in-hospital death, and its level was significantly higher among non-survivors than survivors (80). However, in the study by Wołowiec et al., patients who reached the composite endpoint of unplanned hospitalization and death for all causes during a 24-month follow-up had lower Cts levels – assessed both before and after physical exertion. Firth coefficient was 6.58 (penalized 95% CI 1.66–21.78, *p* = 0.003) (79). Again, the divergent data could result from different study populations: Wołowiec et al. enrolled only hemodynamically stable patients (79), while in the CATSTAT-HF Study patients with acute decompensation of chronic HF were included (80), and more than half of the patients (55.9%) in the study by Peng et al. study were classified as NYHA III-IV (78).

#### Catestatin in Other Diseases Affecting the Cardiovascular System

In the scope of CVD, apart from studies on the role of Cts in HT, CAD, and HF, few other have been published.

Sun et al. studied hemodialyzed patients and demonstrated that plasma Cts levels equal to or greater than the mean (1.9 ng/ml) were associated with higher cardiac death risk (RR 6.13, 95% CI 2.54, 18.45) during a 36-month follow-up. Moreover, there was no such association for non-cardiac death (RR 1.29, 95% CI 0.70, 2.85) (81).

Izci et al. showed that plasma Cts levels were higher in patients with acute pulmonary embolism than in control subjects. Also, there was a positive correlation between Cts and right ventricular dysfunction, and between Cts and Simplified Pulmonary Embolism Severity Index(±0.581, *p* < 0.001), a score used to estimate 30-day mortality in patients diagnosed with non-high-risk acute pulmonary embolism. Furthermore, a Cts cut-off level of 31.2 ng/ml predicted mortality with a sensitivity of 100% and specificity of 52.6% (AUC = 0.883, 95% CI: 0.689–0.921) (82).

In preeclampsia patients, plasma Cts was significantly elevated compared to controls: 0.29 ± 0.096 vs. 0.183 ± 0.072 (*p* < 0.001) (83), and correlated positively with systolic and diastolic BP, urea, creatinine and uric acid levels (83).

Stress may account for- at least in part-, the extent of sympathetic nervous activation, deleteriously affects the immune and coagulation systems, increasing the risk of CVDs. Interestingly, it was demonstrated recently that plasma CgA correlated positively with effort, overcommitment, and effort-reward imbalance (r = 0.267, 0.319, and 0.304, respectively, *p* < 0.001 for all three), and negatively with reward (r = −0.237, *p* < 0.001). Plasma CgA-to-Cts ratio was also associated with work stress in a manner similar to CgA (84).

#### Summary of Clinical Studies

Clinical studies regarding Cts in CAD and HF are summarized in Table 1. It must be highlighted that CAs release causes an increase in Cts, which changes dynamically. Low levels of Cts may play a pathogenic role in cardiac ischemia (74). Research indicates that Cts is involved in the course of CAD as well as HF, and, possibly, its concentration may be applied in monitoring.

## Role of Catestatin in Metabolic Disorders and Atherosclerosis

Metabolic disorders such as obesity, insulin resistance and type 2 diabetes are associated with CVDs. Obesity with an abnormal lipid profile may lead to insulin resistance and is associated with higher cardiovascular risk. In turn, insulin resistance correlates strongly with cardiovascular pathology and is a powerful predictor of future development of type 2 diabetes, an independent risk factor for CVDs (101).

Dysregulated immune system is involved in the pathogenesis of these widely prevalent metabolic disorders. The interdependence of adverse systemic metabolic conditions and immune responses gave rise to the term “immunometabolism,” which is currently also used to describe pathologic reprogramming of immune cells not only in the spectrum of metabolic syndrome, but also other diseases (e.g., neoplasms and autoimmunity). Here, the former meaning of immunometabolism was adopted.

### *In vitro* and *in vivo* Animal Studies

Based on *in vitro* and *in vivo* studies with rodent models, Cts plays a role in the crosstalk between the immune and metabolic systems (15), in particular, in the development of atherosclerosis (19). Cts may act as an anti-atherogenic and anti-inflammatory peptide that reduces leukocyte-endothelium interaction by activating angiotensin-converting enzyme-2 and suppressing tumor necrosis factor-α-elicited expression of inflammatory cytokines and adhesion molecules (19); development of atherosclerosis was attenuated by Cts treatment in apolipoprotein E knockout mice fed a high-fat diet (a mouse model of atherosclerosis) (19) (Figure 1). Further, after vascular injury, Cts increased the expression of *Mrc1*, a gene encoding an anti-inflammatory peptide, and prevented macrophage-driven atherosclerosis (75). Presumably, anti-inflammatory actions of Cts partly depend on the regulation of chemotaxis (102). *In vitro* and *in vivo* (rodent models), Cts counteracted chemoattraction of monocytes and neutrophils by inflammatory chemokines (102), likely contributing to reduced immune infiltration in the heart (21, 75). Regulation of chemotaxis by Cts is complex and naturally occurring Cts variants differ in their chemotactic properties (103, 104), which may influence the propensity for CVD.

The liver plays both a critical role in metabolism, and serves as an important site of immune regulation as it contains resident immune cells as well as synthesizes a number of inflammatory proteins (105). Both tissue-resident (Kupffer cells) and recruited macrophages contribute to an inflammatory state of the liver, e.g., in obesity-induced insulin resistance and type 2 diabetes. Using a rodent model, Ying et al. showed that Cts may inhibit the function and infiltration of macrophages in the liver, which suppresses hepatic gluconeogenesis and improves insulin sensitivity (20). Moreover, the authors demonstrated that treatment of diet-induced obese mice with Cts elicited beneficial changes, including decreased plasma lipids and insulin as well as hepatic lipid content, attenuated expression of gluconeogenic and proinflammatory genes, and increased expression of anti-inflammatory genes in both Kupffer cells and recruited monocyte–derived macrophages in the liver (20) (Figure 1). Furter *in vivo* research on both diet-induced obese and normal chow diet mice showed that Cts decreased obesity-induced endoplasmic reticulum dilation in hepatocytes and macrophages, and enhanced insulin sensitivity in mammalian cells (106).

Latest research *in vivo* on a rodent Cts-KO model has shown that Cts directly promotes hepatic glycogen synthesis, reduces gluneogenesis and glycogenolysis, as well as enhances downstream insulin signaling (23). Positive effects of Cts were observed in Chga-KO mice too, which – despite regular chow diet - were obese, and had increased plasma CAs, leptin, adiponectin, and ketone bodies at baseline (22). Moreover, compared to WT controls, Chga-KO mice exhibited higher glucose-stimulated insulin secretion, which was likely caused by changes in secretory vesicles and mitochondria observed in CgA-deficient β-cells (107). In this model, treatment with Cts lowered circulating CAs and leptin as well as reduced adipose tissue by about 25% resulting in a lean phenotype, increased lipolysis, enhanced fatty acid oxidation and assimilation into lipids in the liver (Figure 1). These beneficial metabolic effects of exogenous Cts were presumed to result from reduction of CA and adiponectin resistance; the effect was mediated by the inhibition of α2 adrenergic signaling (22) (Figure 1). In addition, Cts improved leptin signaling (determined by phosphorylation of AMPK and Stat3 in Chga-KO mice) and peripheral leptin sensitivity in both diet-induced obese mice and in leptin-deficient *ob/ob* mice (22) (Figure 1). In another model with genetically modified rodents, Cts was found to restore natrium-glucose transporter 1 (SGLT1) expression and abundance as well as intestinal turnover in double knock-out leptin receptor b mice, an experimental model of obesity, type 2 diabetes with hyperleptinemia. The effect was possibly mediated by antagonistic binding of Cts to the leptin receptor a (108).

To sum up, it is increasingly clear that Cts is vital for maintaining metabolic homeostasis, which is partly achieved by affecting the immune system. Based on *in vitro* and *in vivo* experimental studies, Cts may inhibit inflammatory response and leukocyte-endothelial cell interactions (19, 21, 75, 102, 103), prevent macrophage-driven atherosclerosis (19, 75), regulate monocyte migration (103), and cytokine production and release (102–104). As a novel regulator of metabolism, in rodents, Cts was shown to help in achieving insulin sensitivity, overcoming endoplasmic reticulum stress (106), reducing adipose tissue by increasing lipolysis, enhancing oxidation of fatty acids, and their assimilation into lipids (22).

### Clinical Studies

So far, only few studies have been conducted in humans on the metabolic role of Cts. Clearly, more research is required in this area.

In a study reported above, O'Connor et al. showed that plasma Cts levels correlated negatively with BMI and plasma leptin concentrations in hypertensive and normotensive subjects (59). Durakoglugil et al. reported that plasma Cts was an independent predictor of high-density lipoprotein cholesterol, and correlated inversely with plasma triglycerides (60). Interestingly, the Ser-364 allele was strongly associated with elevated plasma triglyceride and glucose levels (30).

Simunovic et al. compared plasma Cts levels in 92 obese children and adolescent with those of 39 healthy (normal weight) controls: it levels were significantly lower in the former (10.03 ± 5.05 vs. 13.13 ± 6.25 ng/mL, *p* = 0.004) (85), and, in addition, lower in the subgroup of obese patients with metabolic syndrome vs. those without and controls (9.02 ± 4.3, 10.54 ± 5.36, and 13.13 ± 6.25 ng/ml, respectively, *p* = 0.008). Moreover, Cts negatively correlated with diastolic BP (r = −0.253, *p* = 0.014), homeostatic model assessment of insulin resistance index (r = −0.215, *p* = 0.037), and high sensitivity C-reactive protein (r = −0.208, *p* = 0.044) (85) (Table 1). In another study, children with obstructive sleep apnea (OSA) had reduced plasma Cts levels (Log Cts in moderate-to-severe OSA: 0.12 ± 0.22 vs. mild OSA: 0.23 ± 0.20 vs. controls: 0.28 ± 0.19; *p* < 0.01) and Cts correlated negatively with apnea-hypopnea index (r = −0.226; *p* < 0.01) as well as mean arterial BP (r = −0.184; *p* < 0.05) (86) (Table 1).

Borovac et al. measured plasma Cts levels in 78 male OSA patients aged 50.3 ± 8.8 years and 51 age-, sex- and BMI-matched control subjects. They demonstrated that Cts serum levels are higher in the former (2.9 ± 1.2 vs. 1.5 ± 1.1 ng/mL, *p* < 0.001) (87). Cts significantly correlated with neck circumference (r = 0.318, *p* < 0.001; β = 0.384, *p* < 0.001) and high-density lipoprotein cholesterol (r = – 0.320, *p* < 0.001; β = – 0.344, *p* < 0.001), as well as apnea-hypopnea index among non-obese obstructive sleep apnea subjects (r = 0.466, *p* = 0.016; β = 0.448, *p* = 0.026) (87) (Table 1).

Evidently, reduced Cts levels seem to be associated with an adverse metabolic profile, including obesity and metabolic syndrome, abnormal lipid concentrations and insulin resistance.

## Conclusions and Perspectives

In conclusion, current data indicate that the endogenous bioactive peptide Cts is a vital regulatory factor of cardiovascular and immunometabolic homeostasis. Possibly, in future, Cts can be used in the diagnosis of CVDs and metabolic disorders as a novel biomarker, which may aid in clinical decision-making. Application of Cts in therapy might potentially mitigate detrimental sympathoexcitatory effects, which underlie cardiovascular and metabolic diseases. It should be highlighted that, so far, studies on Cts have been carried out mainly on animal models. More research is required to take advantage of beneficial effects of Cts in clinical practice.

## Author Contributions

EZ and PK review the literature, and EZ wrote the first draft of the manuscript. PK and EZ wrote sections of the manuscript. KS carried out critical interpretations. All authors contributed to manuscript revision, read, and approved the submitted version.

## Conflict of Interest

The authors declare that the research was conducted in the absence of any commercial or financial relationships that could be construed as a potential conflict of interest.

## Publisher's Note

All claims expressed in this article are solely those of the authors and do not necessarily represent those of their affiliated organizations, or those of the publisher, the editors and the reviewers. Any product that may be evaluated in this article, or claim that may be made by its manufacturer, is not guaranteed or endorsed by the publisher.

## Abbreviations

Akt: Protein kinase B
AMI: acute myocardial infarction
BP: blood pressure
Ca^2+^: calcium ions
CAD: coronary artery disease
CA: catecholamine
CgA: Chromogranin A
Cts-KO: catestatin knockout
CVD: cardiovascular disease
Chga-KO: Chromogranin knockout
Cts: catestatin
hCgA: human CgA
HF: heart failure
HT: hypertension
I/R injury: ischemia/reperfusion injury
nAChR: nicotinic acetylcholine receptor
NO: nitric oxide
nNOS: neuronal nitric oxide synthase
NYHA: New York Heart Association
OSA: obstructive sleep apnea
PMNs: Polymorphonuclear neutrophils
WT: wild type.
